# Treatment characteristics of patients with hereditary transthyretin amyloidosis: a cohort study

**DOI:** 10.1186/s13023-024-03198-7

**Published:** 2024-05-08

**Authors:** Taha N. Qarni, Felipe J. S. Jones, Brian Drachman, Sami Khella, Janice Pieretti, Nicolas Sarmiento Bustamante, Chafic Karam

**Affiliations:** 1 Department of Neurology, Hospital of the University of Pennsylvania, University of Pennsylvania, 3400 Spruce St, 3 Gates, Philadelphia, PA 19104 USA; 2 Department of Cardiology, University of Pennsylvania, Philadelphia, PA USA; 3 Penn Amyloidosis Center, University of Pennsylvania, Philadelphia, PA USA

**Keywords:** Transthyretin amyloidosis, Tafamidis, Inotersen, Diflunisal, Patisiran, Vutrisiran

## Abstract

**Background:**

There are novel medications approved for the treatment of hereditary transthyretin amyloidosis (ATTRv), classified as transthyretin (TTR) stabilizers or gene silencers. While many patients may be on both classes of medications, there is no data available on the safety and efficacy of combination therapy.

**Objectives:**

To describe ATTRv patient and TTR-targeted therapy characteristics in a US cohort, and compare outcomes with combination therapy versus monotherapy.

**Methods:**

We performed a retrospective cohort study with electronic health record data of patients with ATTRv seen at a single institution between January 2018 and December 2022. We collected data on symptomatology, gene mutation, disease severity, ATTRv treatment, hospitalizations, and mortality.

**Results:**

One hundred sixty-two patients with ATTRv were identified. The average age at diagnosis was 65 years. 86 patients (53%) had the V122I variant. 119 patients were symptomatic, of whom 103 were started on ATTRv-specific treatment. 41 patients (40%) had cardiomyopathy only, and 53 (51%) had a mixed phenotype of cardiomyopathy and neuropathy. 38 patients (37%) received therapy with both a gene silencer and protein stabilizer. 9 patients (15%) in the monotherapy group had two or more cardiac hospitalizations after starting treatment, compared to 3 patients (9%) on combination therapy (*p*=0.26). The adjusted hazard ratio of all-cause mortality for the patients on combination therapy compared to monotherapy was 0.37 (0.08-1.8, *p*=0.21).

**Conclusions:**

While the efficacy is unproven, over one-third of patients with ATTRv are on both a stabilizer and a silencer. There were no safety issues for combination therapy. There was a trend towards improved hospitalizations and survival in patients in the combination group but this was not statistically significant. Larger studies with longer follow-up are necessary to determine benefit of combination therapy.

## Key summary points

• A large proportion of patients with ATTRv are on both a silencer and stabilizer despite the lack of evidence of benefit.

• In our patients’ population, the combination therapy was found to be safe.

• No clear benefit was found between patients on monotherapy versus combination therapy. However, a trend of less hospitalization and mortality was observed in those patients on combination therapy.

## Introduction

Hereditary transthyretin amyloidosis (ATTRv) is a progressive, genetic condition caused by pathological variants in the transthyretin (TTR) [1, 2]. There have been recent developments of novel therapeutic options that target various stages of the amyloidogenic cascades. Broadly speaking, these are divided into protein stabilizers and gene silencers.

Protein stabilizers bind to and stabilize tetrameric TTR, preventing degradation into pathogenic TTR monomers [3, 4]. The two stabilizers most commonly used are Tafamadis and Diflunisal. Tafamidis is approved for treatment of both cardiomyopathy and neuropathy in Europe and other countries, although the Food and Drug Administration (FDA) indication remains limited to ATTR cardiomyopathy in the United States [5, 6]. Diflunisal is used off-label for both ATTR amyloid cardiomyopathy and neuropathy [7–9].

The gene silencers include Inotersen, Patisiran, Vutrisiran, and Eplotersen. Inotersen is an antisense oligonucleotide inhibitor of hepatic production of transthyretin protein [10]. Patisiran is an RNA interference agent that targets the 3’ untranslated region of TTR mRNA to reduce hepatic production [10]. Both were approved by the FDA for the treatment of ATTRv-associated neuropathy in 2018 after two randomized controlled trials (RCTs) demonstrated benefit in reducing circulating levels of TTR and neuropathy impairment and improving quality of life compared to placebo [11, 12]. Neither of these studies primarily evaluated the effects on cardiomyopathy. However, a subgroup analysis for Patisiran did demonstrate improved cardiac biomarkers, and a subsequent smaller open-label study of Inotersen also demonstrated a benefit in cardiomyopathy [13, 14]. Vutrisiran was approved for ATTRv-associated peripheral neuropathy in 2022 [15]. Epolotersen is not yet approved but results from its clinical trial were positive. Since in the US many patients will have both cardiac and neurologic manifestations of their disease, they technically qualify to be on both a gene silencer and protein stabilizer [3, 4]. Nevertheless, there is scarce data on the safety and efficacy of the combination gene silencers and protein stabilizers in this population.

The objectives of this study are to describe the clinical and treatment characteristics of a cohort of patients with ATTRv, and to compare the safety and efficacy of combination therapy (i.e., concurrent use of a protein stabilizer plus a gene silencer) versus monotherapy (i.e., use of either a protein stabilizer or a gene silencer) in this population.

## Methods

The University of Pennsylvania IRB approved this study. We performed a retrospective cohort study using electronic health record data of patients seen at the University of Pennsylvania between January 1^st^, 2018 and December 31^st^, 2022. We included data from all adult patients with a known pathogenic TTR variant on genetic testing. This study was approved by the University of Pennsylvania institutional review board and followed the Strengthening the Reporting of Observational Studies in Epidemiology (STROBE) reporting guideline [16].

### Data collection

Two authors (T.Q. and N.S.B.) retrospectively collected data from electronic health records using a secure, HIPAA-compliant data abstraction tool. Data used to describe the sample included age, age at ATTRv diagnosis, sex (female or male), TTR genotype, presence of ATTRv-related symptoms (symptomatic or asymptomatic), type of symptom (cardiomyopathy, arrhythmia, carpal tunnel syndrome, peripheral neuropathy, and/or autonomic neuropathy), year of onset for each symptom, diagnosis delay (time from symptom onset to diagnosis in years), presence of alternative causes of neuropathy (alcohol use disorder, diabetes, vitamin B12 deficiency), New York Heart Association (NYHA) functional class at baseline, cardiac biomarkers at baseline (troponin levels, NT-proBNP, left ventricular ejection fraction, left ventricular internal diameter end diastole, interventricular septum thickness, longitudinal strain), and neuropathy impairment scores at baseline.

### Exposure and follow-up

We collected prescription data on 5 ATTRv medications (Tafamidis, Diflunisal, Inotersen, Patisiran, and Vutrisiran). We defined treatment type as a time-varying exposure comprising combination therapy (i.e., concurrent use of a gene silencer and a protein stabilizer) or monotherapy (i.e., use of either a gene silencer or a protein stabilizer). The follow up period began at initiation of any treatment for ATTRv until death or December 31^st^, 2022.

### Outcomes

We defined two cardiac outcomes: incidence of all-cause mortality and number of hospitalizations for heart failure during the follow up period. We compared these outcomes in the subset of patients who had ATTRv-related cardiomyopathy.

### Statistical analysis

We described the sample characteristics using median (interquartile range, IQR) for nonparametric continuous variables and absolute frequency (percentages, %) for categorical variables.

The characteristics of patients who were treated with monotherapy versus combination therapy were compared using the Wilcoxon rank-sum test and Chi-square tests for nonparametric continuous and categorical variables, respectively. The association between combination therapy and all-cause mortality was examined using Kaplan-Meier analysis and a multivariate time-varying Cox-proportional hazards model. Age and TTR variant were defined a priori as covariates to be included in the multivariate analysis. Other covariants for inclusion (i.e. cardiac biomarkers) were unable to be selected given the amount of missing data and small number of outcomes. The association between combination therapy and number of hospitalizations for heart failure was assessed using the Chi-square test. A *p*-value <.05 was considered statistically significant based on two-sided tests. Stata 16 (StataCorp, 2019. Stata Statistical Software: Release 16. College Station, TX) was used to perform statistical analyses.

## Results

### Patient demographics

The charts of 162 patients were reviewed, all of whom had a genetically confirmed TTR variant. The most common mutations amongst the entire cohort were V1221 (86 patients), T60A (31 patients), and V30M (17 patients) (Table 1). Of the 162 patients included in the initial chart review, 119 were symptomatic from ATTRv. The median age of symptomatic patients at diagnosis was 70 years (IQR 62-75) compared to 52.5 years (IQR 43-57) for the asymptomatic group. A larger portion of symptomatic patients were male compared to the asymptomatic group (68.9% vs 34.9%).

**Table 1:** Characteristics of all patients with ATTRv (*n*=162)

**Characteristic**	**Total** **(*N* = 162)**	**Symptomatic** **(*N* = 119)**	**Asymptomatic** **(*N* = 43)**
Median age at diagnosis, years (IQR)	65 (55-74)	70 (62-75)	52.5 (43-57)
Male sex, n (%)	97 (59.9)	82 (68.9)	15 (34.9)
TTR genotype, n (%)			
V122I	86 (53.1)	68 (57.1)	18 (41.9)
T60A	31 (19.1)	20 (16.8)	11 (25.6)
V30M	17 (10.5)	11 (9.2)	6 (13.9)
Phe64	12 (7.4)	8 (6.7)	4 (9.3)
Others	16 (9.9)	12 (10.0)	4 (9.3)

*Abbreviations: N or n* total number, *IQR* Interquartile range, *%* percentage

### Characteristics of symptomatic patients

Among the 119 symptomatic patients studied, the median time to diagnosis after symptom onset for patients was 3 years (IQR 1-8 years) (Table 2). 103 patients (86.5%) were treated with specific ATTR medications. Sixteen patients were not treated. Of these, eight were lost to follow-up, 3 had advanced disease or died before initiation of treatment, and 5 were pending initiation of treatment. The median age of patients who were treated was 71 years (IQR 63-75 years). The median age of patients who were not treated was 59 years (IQR 54-73). The most common symptoms at onset of disease were: carpal tunnel syndrome (44 patients, 37%), heart failure (36 patients, 30.1%), and peripheral neuropathy (20 patients, 16.8%). Twenty seven (22.7%) patients had co-existing diabetes (Table 2).

**Table 2:** Characteristics of all symptomatic patients by treatment status (*n*=119)

**Characteristic**	**Symptomatic** **(*N* = 119)**	**Treated** **(*N* = 103)**	**Not treated** **(*N*= 16)**
Median age at symptom onset, years (IQR)	64 (56-72)	66 (57-73)	54 (48-63)
Median age at diagnosis, years (IQR)	70 (62-75)	71 (63-75)	59 (54-73)
Median diagnosis delay, years (IQR)	3 (1-8)	3 (1-8)	4 (1-13)
Male sex, n (%)	82 (68.9)	70 (68.0)	12 (75.0)
Alcohol use disorder, n (%)	7 (5.9)	5 (4.8)	2 (12.5)
Diabetes, n (%)	27 (22.7)	22 (21.3)	5 (31.2)
TTR genotype, n (%)			
V122I	68 (57.1)	56 (53.4)	12 (75.0)
T60A	20 (16.8)	19 (18.4)	1 (6.3)
V30M	11 (9.2)	11 (10.7)	0 (0)
Phe64	8 (6.7)	8 (7.8)	0 (0)
Others	12 (10.0)	9 (8.7)	3 (18.7)

*Abbreviations: N or n* total number, *IQR* Interquartile range, *%* percentage

Of the symptomatic patients, 47 had cardiac symptoms only, 13 had neurological symptoms only, and 59 patients had both cardiac and neurological symptoms (mixed symptomatology) (Table 3). Patients with isolated neurological symptoms were younger at the time of diagnosis compared with those with cardiac symptoms (isolated or mixed), with median ages of 52 years, 74 years, and 67 years, respectively. Most patients with isolated cardiac symptoms carried the V122I mutations (42 patients, 89.4%) (Table 3).

**Table 3:** Characteristics of symptomatic patients by symptom type (*n*=119)

**Characteristic**	**Symptomatic** **(*N* = 119)**	**Both** **(*N*=59)**	**Cardiac only** **(*N* = 47)**	**Neuro only** **(*N*=13)**
Median age at symptom onset, years (IQR)	64 (56-72)	62 (56-68)	70 (65-74)	50 (44-56)
Median age at diagnosis, years (IQR)	70 (62-75)	67 (62-74)	74 (71-78)	52 (47-58)
Median diagnosis delay, years (IQR)	3 (1-8)	3 (1-7)	3 (1-9)	1 (0-5)
Male sex, n (%)	82 (68.9)	46 (78.0)	32 (68.1)	4 (30.1)
Alcohol use disorder, n (%)	7 (5.9)	4 (6.8)	2 (4.2)	1 (7.7)
Diabetes, n (%)	27 (22.7)	14 (23.7)	12 (25.5)	1 (8.3)
TTR genotype, n (%)				
V122I	68 (57.1)	23 (39.0)	42 (89.4)	3 (23.1)
T60A	20 (16.8)	16 (27.1)	2 (4.3)	3 (23.1)
V30M	11 (9.2)	7 (11.8)	1 (2.1)	2 (15.4)
Phe64	8 (6.7)	5 (8.5)	0 (0)	3 (23.1)
Others	12 (10.0)	8 (13.5)	2 (4.2)	2 (15.4)
First symptom at onset, n (%)				
Carpal tunnel syndrome	44 (37.0)	19 (32.2)	21 (44.7)	4 (30.8)
Heart failure	36 (30.1)	15 (25.4)	21 (44.7)	0 (0.0)
Arrhythmia	12 (10.1)	7 (11.9)	5 (10.6)	0 (0.0)
Neuropathy, somatic	20 (16.8)	14 (23.7)	0 (0.0)	6 (46.1)
Neuropathy, autonomic	6 (5.0)	4 (6.8)	0 (0.0)	2 (15.4)
Treated, n (%)	103 (86.5)	53 (89.8)	41 (87.2)	9 (69.2)
Treatment type, n (%)				
Combination therapy	38 (36.9)	34 (64.1)	0 (0)	4 (44.4)
Monotherapy	65 (63.1)	19 (35.8)	41 (100)	4 (44.4)
Drug, n (%)				
Tafamidis monotherapy	54 (52.4)	13 (24.5)	41 (100)	0 (0)
Diflunisal monotherapy	5 (4.6)	1 (1.9)	0 (0)	4 (44.4)
Patisiran monotherapy	4 (3.9)	4 (7.5)	0 (0)	0 (0)
Inotersen monotherapy	2 (1.9)	1 (1.9)	0 (0)	1 (7.7)
Tafamidis and Patisiran	19 (18.4)	19 (35.8)	0 (0)	0 (0)
Tafamidis and Inotersen	3 (2.9)	3 (5.7)	0 (0)	0 (0)
Tafamidis and Vutisiran	2 (1.9)	2 (3.8)	0 (0)	0 (0)
Diflunisal and Patisiran	11 (10.7)	8 (15.1)	0 (0)	3 (33.3)
Diflunisal and Inotersen	3 (2.9)	2 (3.8)	0 (0)	1 (7.7)

*Abbreviations: N or n* total number, *IQR* Interquartile range, *%* percentage

### Patient characteristics based on treatment type

**Fig. 1:**
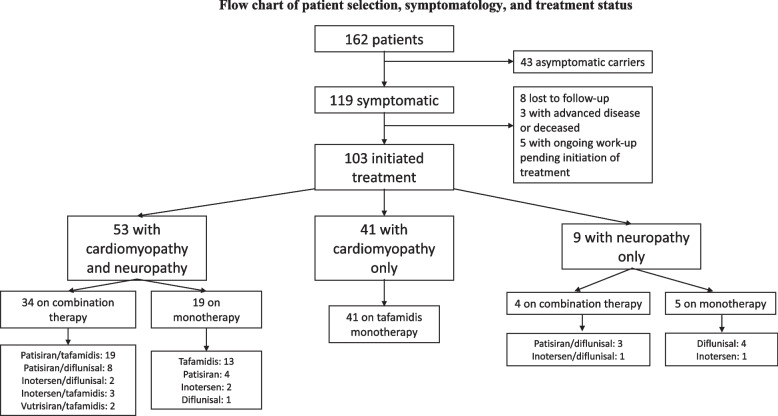
Flow chart of patient selection, symptomatology, and treatment status. Figure 1 demonstrates a flow chart of patients included in this retrospective study. One hundred sixty-two patients were identified who had genetically-proven ATTRv. Of these, 119 were symptomatic, and 103 of those patients were started on treatment. Fifty-three patients had a mixed phenotype of both cardiomyopathy and neuropathy, 41 had cardiomyopathy only, and 9 had neuropathy only. The flow chart depicts treatment regimens (monotherapy or combination therapy) stratified by symptom type

Of the 103 patients who received treatment for ATTRv, 65 (63%) were maintained on monotherapy with either a stabilizer (Tafamidis, Diflunisal) or silencer (Inotersen, Patisiran, Vutrisiran). Thirty eight (37%) patients were treated with combination therapy with both a silencer and stabilizer. Amongst the 59 patients with both cardiac and neurological symptoms, 34 (64.1%) were treated with combination therapy (Fig. 1). The most common combinations amongst patients with both cardiac and neurological symptoms were Tafamidis plus Patisiran (19 patients, 35.8%), and Diflunisal plus Patisiran (8 patients, 15.1%); the other 7 patients were on a combination of either Inotersen or Vutrisiran with Tafamidis or Diflunisal. The remaining 4 patients treated with combination therapy had only neurological symptoms. These patients were all on a combination of Diflunisal plus a silencer. Nineteen patients had both cardiac and neurological symptoms and were technically eligible for combination therapy but were only on monotherapy. The most common reasons for not being on combination therapy in this group were: loss of neurologic follow-up (5 patients), alternative causes of neuropathy such as diabetes or chemotherapy (4 patients), and therapy side effects (4 patients). In the latter group, two were started on Diflunisal but it was stopped due to nausea and bruising, respectively. One patient developed thrombocytopenia with Inotersen, and another developed worsening hypertension with Tafamidis. Of the patients with neuropathy, 4 were not on a silencer because of mild neurological symptoms not proven to be due to amyloid deposition (negative or pending biopsies). The median time patients were on monotherapy at the time of data extraction was 38 months (IQR 20-45), and the median time for combination therapy was 27 months (IQR 15-52) (Fig. 1).

The characteristics for all patients with cardiomyopathy are displayed in Table 4. Those on combination therapy were younger at initiation of treatment than those on monotherapy, with a median age of 67 years (IQR 60-72) compared to 75 years (IQR 72-78). They also had a lower median age at symptom onset (60 years vs 69 years respectively, *p*<0.05) and at diagnosis (66 years vs 74 years respectively, *p*<0.05). Patients on monotherapy were more likely to carry the V122I variant than those on combination therapy (78.3% vs 23.5%). There was no difference between patients on combination therapy versus patients on monotherapy in terms of the gender, and the concurrent presence of diabetes or alcohol use disorder.

**Table 4:** Characteristics of patients with any cardiac symptoms based on treatment type (*n*=94)

**Characteristic**	**Monotherapy** **(*N* = 60)**	**Combination** **(*N* = 34)**	***p*-values**
Median age at start of treatment, years (IQR)	75 (72-78)	67 (60-72)	< 0.001
Median age symptom onset, years (IQR)	69 (63-73)	60 (51-67)	< 0.001
Median age at diagnosis, years (IQR)	74 (70-77)	66 (61-70)	< 0.001
Male sex, n (%)	42 (70.0)	26 (76.5)	0.500
Diabetes, n (%)	17 (28.3)	5 (14.7)	0.134
Alcohol use disorder, n (%)	3 (5.0)	1 (2.9)	0.635
TTR genotype, n (%)			< 0.001
V122I	47 (78.3)	8 (23.5)	
Others	13 (21.7)	26 (76.5)	
Baseline NYHA^a^			0.095
I	9 (19.6)	5 (20.8)	
II	20 (43.5)	15 (62.5)	
III	17 (37.0)	3 (12.5)	
IV	0 (0)	1 (1.4)	
Baseline median EF (IQR)^b^	42 (30-52)	54 (37-61)	0.032
Baseline median NT-proBNP (IQR)^c^	2963 (1351-4948)	1528 (387-2388)	0.078
Median follow up, months (IQR)	23.6 (15.7-40.0)	40.3 (18.7-75.4)	0.012

*Abbreviations: N or n* total number, *IQR* Interquartile range, *%* percentage

^a^NYHA only available for 70 patients (25% missing)

^b^TTE only available for 66 patients (29% missing)

^c^BNP only available for 57 patients (38% missing)

There was a higher proportion of patients on monotherapy who had a NYHA class of 3 or 4 compared to those on combination therapy (37.0% vs 13.9%), but this difference was not statistically significant (*p*=0.09). Patients on combination therapy had a higher ejection fraction (54% vs. 42%, *p* <0.05) and lower NT-proBNP (1528 vs. 2963, *p*=0.07) before treatment initiation compared to patients on monotherapy. Patients on combination therapy had a longer median follow-up time compared to those on monotherapy (40.3 months vs. 23.6 months, *p*<0.05).

### Association of combination therapy with all-cause mortality and hospitalizations for heart failure

**Fig. 2:**
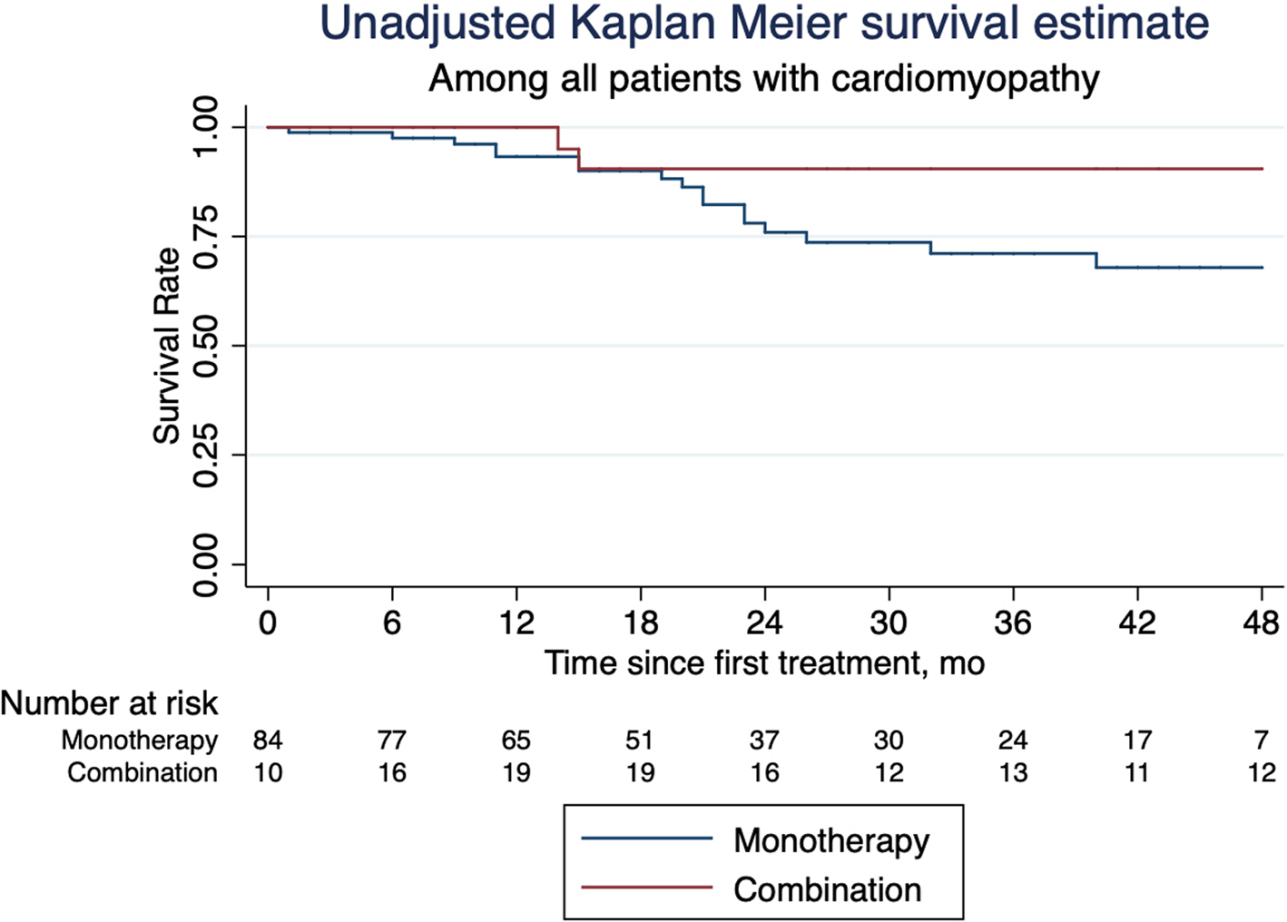
Unadjusted Kaplan Meier survival curve amongst all patients with cardiomyopathy (*n*=94). Figure 2 depicts an unadjusted Kaplan-Meier survival curve amongst all patients with cardiomyopathy comparing combination therapy versus monotherapy, calculated using a multivariate time-varying Cox-proportional hazards model

Thirty-eight patients (46.7%) on monotherapy had one or more hospitalizations for cardiac reasons after treatment initiation compared to 10 patients (29.4%) of the combination group (*p*=0.26) (Table 5). There were 20 incident deaths during the follow up period, 18 in the monotherapy group and two in the combination therapy group. The crude incidence rate for all-cause mortality per 1000 person-months (PM) was 8.2 (95% CI 5.2-13.1) among patients treated with monotherapy and 1.8 (95% CI 0.4-7.2) among patients treated with combination therapy. The unadjusted Kaplan-Meier survival curve is depicted in Fig. 2. After adjustment for age and TTR variant type, the association between combination therapy and all-cause mortality was not statistically significantly different when compared to the monotherapy group (HR 0.37, 95% CI 0.08-1.8, *p* = 0.217).

**Table 5:** Hospitalizations for heart failure in patients with cardiomyopathy (*n*=94)

**Outcomes**	**Monotherapy** **(*N* = 60)**	**Combination** **(*N* = 34)**	***p*-values**
Hospitalization, n (%)			0.260
0	32 (53.3)	24 (70.6)	
1	19 (31.7)	7 (20.6)	
2+	9 (15.0)	3 (8.8)	

*Abbreviations: N or n* total number, *IQR* Interquartile range, *%* percentage

## Discussion

This study is a single site cohort of symptomatic ATTRv patients in the United States demonstrating that a significant proportion of patients (about one-third) are treated with combination therapy (stabilizers and silencers). While this approach is not backed by data and likely increases overall cost of caring for patients, it is not unreasonable given the underlying pathophysiology and drug mechanism of action (TTR silencers suppress production by an average of 80% and the stabilizers can stabilize the rest of the pathologic serum TTR). The average age at diagnosis as well as ATTRv mutation prevalence and phenotype (predominantly cardiomyopathy) were similar to what was observed in prior studies in the US population, thus supporting the generalizability of this cohort [17]. The reduced diagnosis delay with average time to diagnosis after any symptom onset of 3 years reflects increased awareness and screening of family members [18].

In our cohort, a majority of patients had both cardiac and neurological manifestations of ATTRv. Most of these patients were on combination therapy. Of all the patients treated for ATTRv, over one-third were on combination therapy. Combination therapy was well-tolerated, as a majority of patients started on combination therapy did not have any limiting side effects. This poses implications for future clinical practice, as determining the safety and efficacy of combination therapy will be of paramount importance to clinicians caring for ATTRv patients, especially with the rapidly evolving treatment landscape and the high cost of both classes of medications.

We attempted to determine whether patients on combination therapy had an effect on cardiac hospitalizations and all-cause mortality compared to patients on monotherapy. This was potentially confounded by a significant difference in baseline cardiac biomarkers, including higher ejection fractions and lower NT-proBNP in the combination therapy group. There was not a statistically significant difference between the two groups with respect to hospitalizations and mortality, although there appeared to be a trend towards improved outcomes in the combination therapy group.

We did not collect data on TTR levels in our cohort. While silencer therapy has been associated with a decreased level of circulating TTR, stabilizer therapy with diflunisal or tafamadis is associated with an increase in TTR levels [11, 12, 19, 20]. Therefore, the utility of monitoring TTR levels as a surrogate for disease activity in patients on combination therapy versus those on monotherapy is unclear.

The limitations involved with this study are many of the limitations inherent with a retrospective chart review. This was a single-center cohort with 4 clinicians (2 cardiologists and 2 neurologists) evaluating the patients, which decreases the generalizability of data. However, personal communication with US amyloid experts suggests similar approach to treatment in patients with mixed phenotypes (i.e., many patients are on both silencers and stabilizers medications). Since the medications have only been approved since 2018 and 2019, there might be a short time frame to capture events such as hospitalizations or mortality, leading to an underpowered study. Due to the retrospective nature of this study and limitations with in-person follow-up due to COVID 19 pandemic, there was significant missing data regarding neurological evaluations and cardiac biomarkers. Furthermore, the majority of our patients had predominantly cardiac phenotype with mild neuropathy resulting in fewer neurological follow-ups and insufficient data for statistically analysis. Given the degree of missing information, NIS data was not analyzed, and thus the effects of treatment status on neurological symptoms could not be assessed.

Longer term, prospective, multicenter evaluation of combination therapy for ATTRv is needed to determine the optimal therapy for the mixed phenotype patients.

## Conclusions

This study demonstrates that a significant number of patients with ATTRv are treated with combination therapy with a gene silencer and proteins stabilizer. Over 50% of patients with a mixed phenotype were on both classes of medications. Combination therapy was well-tolerated, with no side effects attributed to being on both classes of medications concurrently. There was no statistical difference in cardiac hospitalizations or all-cause mortality amongst patients on combination therapy versus monotherapy, but this was confounded by decreased cardiac severity at baseline for the combination therapy group. The study was also limited by a large amount of missing data and small time of follow-up.

Although this study failed to demonstrate a statistical difference in cardiac hospitalizations or all-cause mortality in patients treated with combination therapy versus monotherapy, it does demonstrate a need for future prospective studies examining this question. This will be of importance for clinicians treating patient with ATTRv as the optimal treatment regimen for this disease is still undetermined, and may continue to evolve with novel therapeutic options in the future. Given the considerable number of patients on combination therapy, the unclear benefit of combination therapy versus monotherapy, and the high costs of these drugs, there is a need for larger studies, with longer follow-up, to determine the optimal treatment regimen for patients with a variety of different symptomatology.

## Data Availability

Not applicable.

## Abbreviations

ATTRv: Hereditary transthyretin amyloidosis
TTR: Transthyretin
FDA: Food and Drug Administration
IQR: Interquartile range
nt-proBNP: N-terminal prohormone of brain natriuretic peptide
NIS: Neuropathy Impairment Score
NYHA: New York Heart Association
RCT: Randomized controlled trial
